# Tumid Lupus Erythematosus: A Rare and Distinctive Variant of Cutaneous Lupus Erythematosus Masquerading as Urticarial Vasculitis

**DOI:** 10.7759/cureus.8305

**Published:** 2020-05-26

**Authors:** Evan Liu, Robert P Daze, Summer Moon

**Affiliations:** 1 Dermatology, Nova Southeastern University, Dr. Kiran C. Patel College of Osteopathic Medicine, Largo, USA; 2 Dermatology, Largo Medical Center, Largo, USA

**Keywords:** tumid lupus, systemic lupus erythematosus, urticarial vasculitis, chronic cutaneous lupus erythematosus

## Abstract

Tumid lupus erythematosus (TLE) is a rare form of chronic cutaneous lupus that has triggered much debate regarding its clinical and histopathological features. It has been classically defined as annular erythematous, succulent, plaques involving the face and trunk that typically are devoid of any papulosquamous features such as scale and follicular plugging. These lesions are a clinical mimicker of other urticarial lesions such as urticarial vasculitis and lymphocytic infiltrate of Jessner. We report a case of TLE presenting in a 49-year-old Caucasian female whose initial clinical presentation was concerning for urticarial vasculitis due to presence of urticarial-like lesions present for approximately three months. Laboratory studies and histopathological correlations confirmed the diagnosis of TLE and the patient was successfully treated with topical corticosteroids.

## Introduction

Tumid lupus erythematosus (TLE) is an uncommon variant of chronic cutaneous lupus erythematosus. It is characterized by photosensitive, erythematous, and edematous lesions affecting the head, neck, and trunk [1]. Clinically TLE may be a diagnostic challenge as it can be difficult to distinguish from lymphocytic infiltrate of Jessner and urticarial vasculitis. Histopathologically, it is very hard to distinguish between TLE and reticular erythematous mucinosis due to the uninvolved epidermis, perivascular infiltrate, and excessive mucin deposition [1]. TLE is typified histologically with a lack of epidermal changes, abundant dermal mucin, and intense perivascular and periadnexal lymphocytic infiltrate [2]. The presence of photosensitivity, distribution of lesions, absence of gross serum abnormalities notably complements, and lack of epidermal changes can aid in the diagnostics. TLE poses as a clinical and histopathological controversy with regard to both reticular erythematous mucinosis and lymphocytic infiltrate of Jessner, and many authors have proposed these entities to exist on a clinicopathological spectrum [1]. The management of TLE typically consists of topical steroids and/or antimalarial medications without scarring or hyperpigmentation. 

## Case presentation

**Figure 1:**
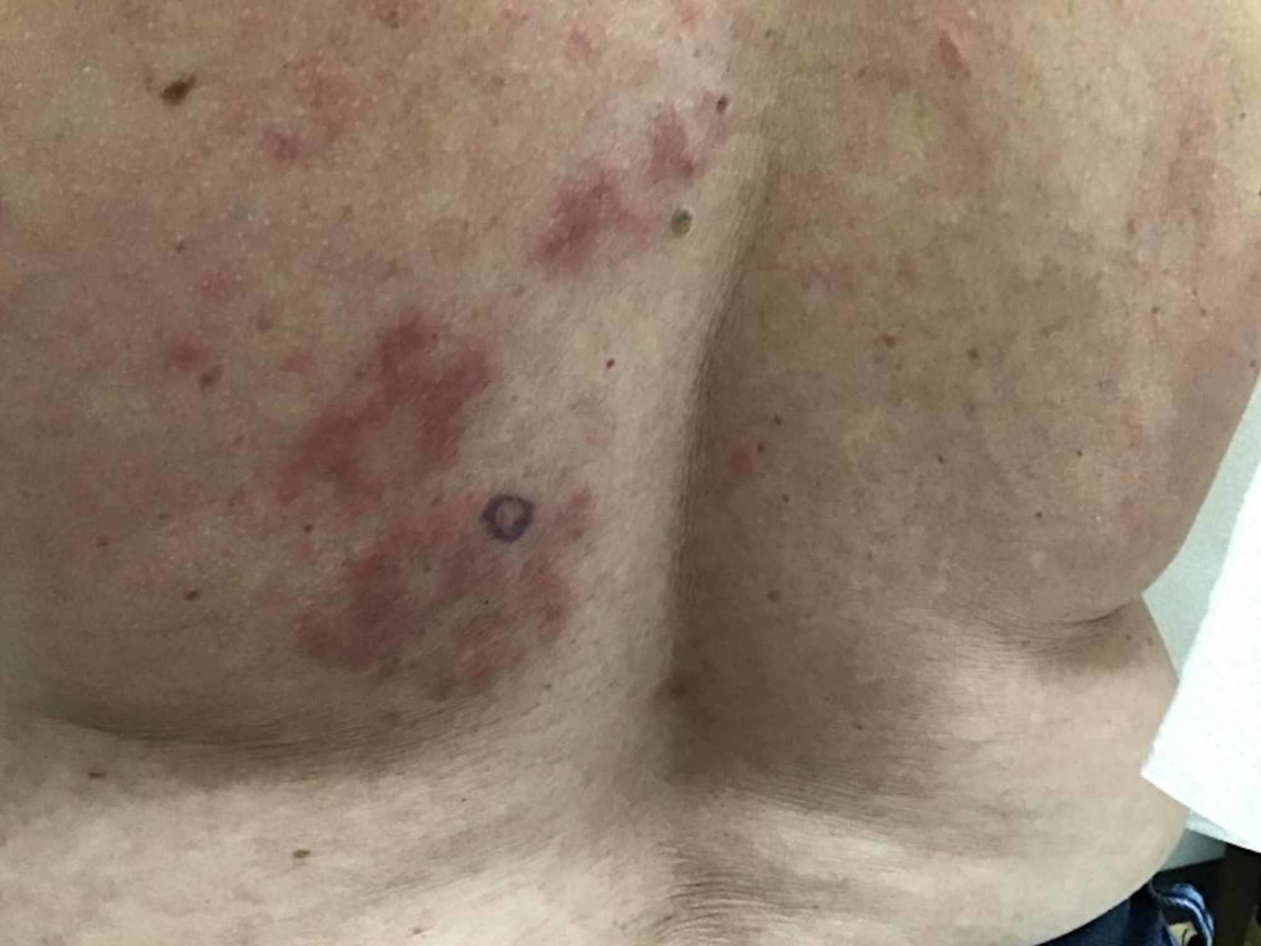
Erythematous, evanescent, edematous plaques of the left inferior medial back. Purple circle marked with surgical pen indicated the area for 4-mm punch biopsy.

**Figure 2:**
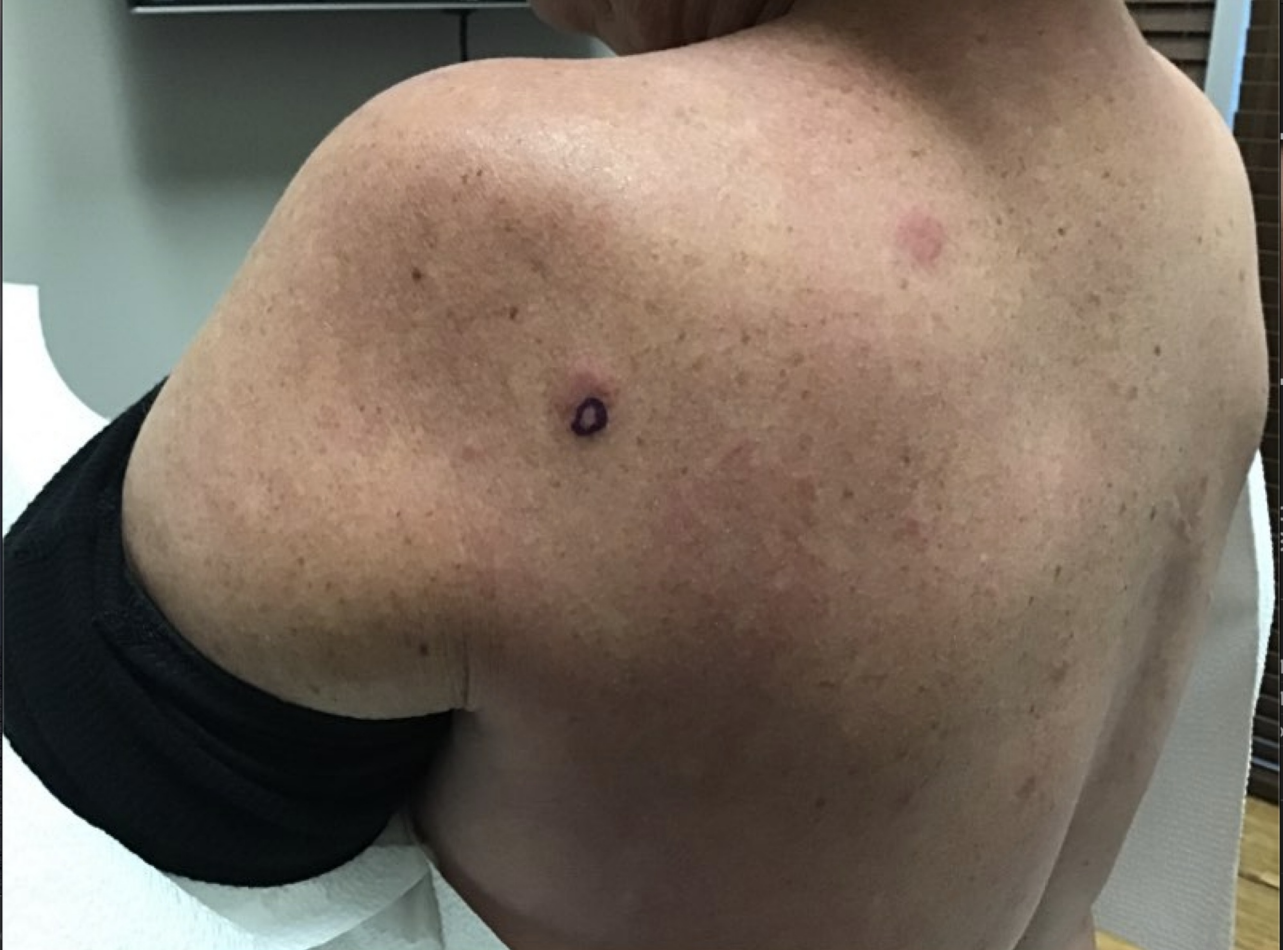
Erythematous, evanescent, edematous plaques of the left superior lateral upper back. Purple circle marked with surgical pen indicated the area for 4-mm punch biopsy.

**Figure 3:**
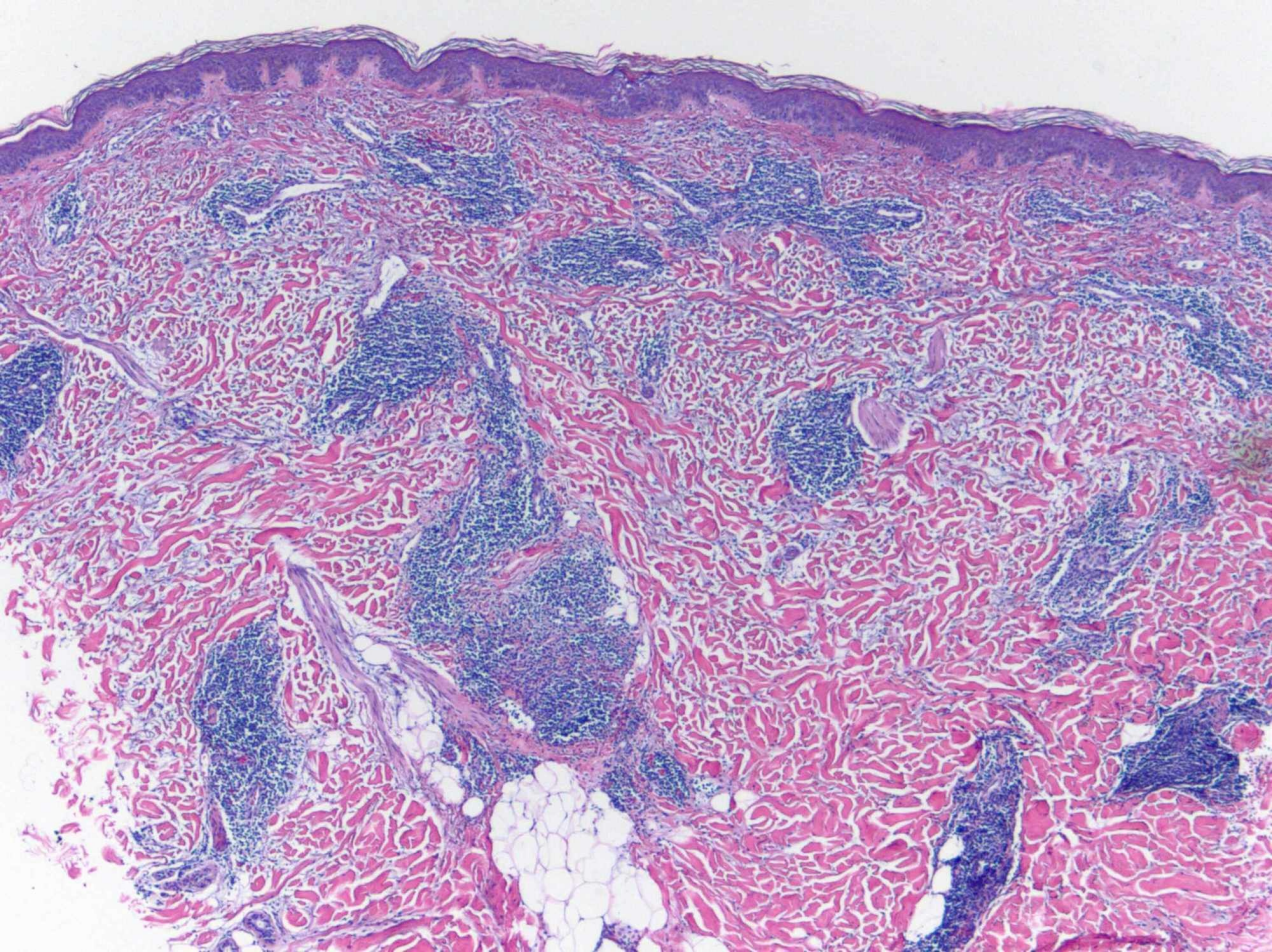
Hematoxylin and eosin stain of 4-mm punch biopsy (4x magnification) of lesion demonstrating superficial and deep perivascular and periadnexal lymphocytic infiltrate with dermal mucin deposition and edema.

**Figure 4:**
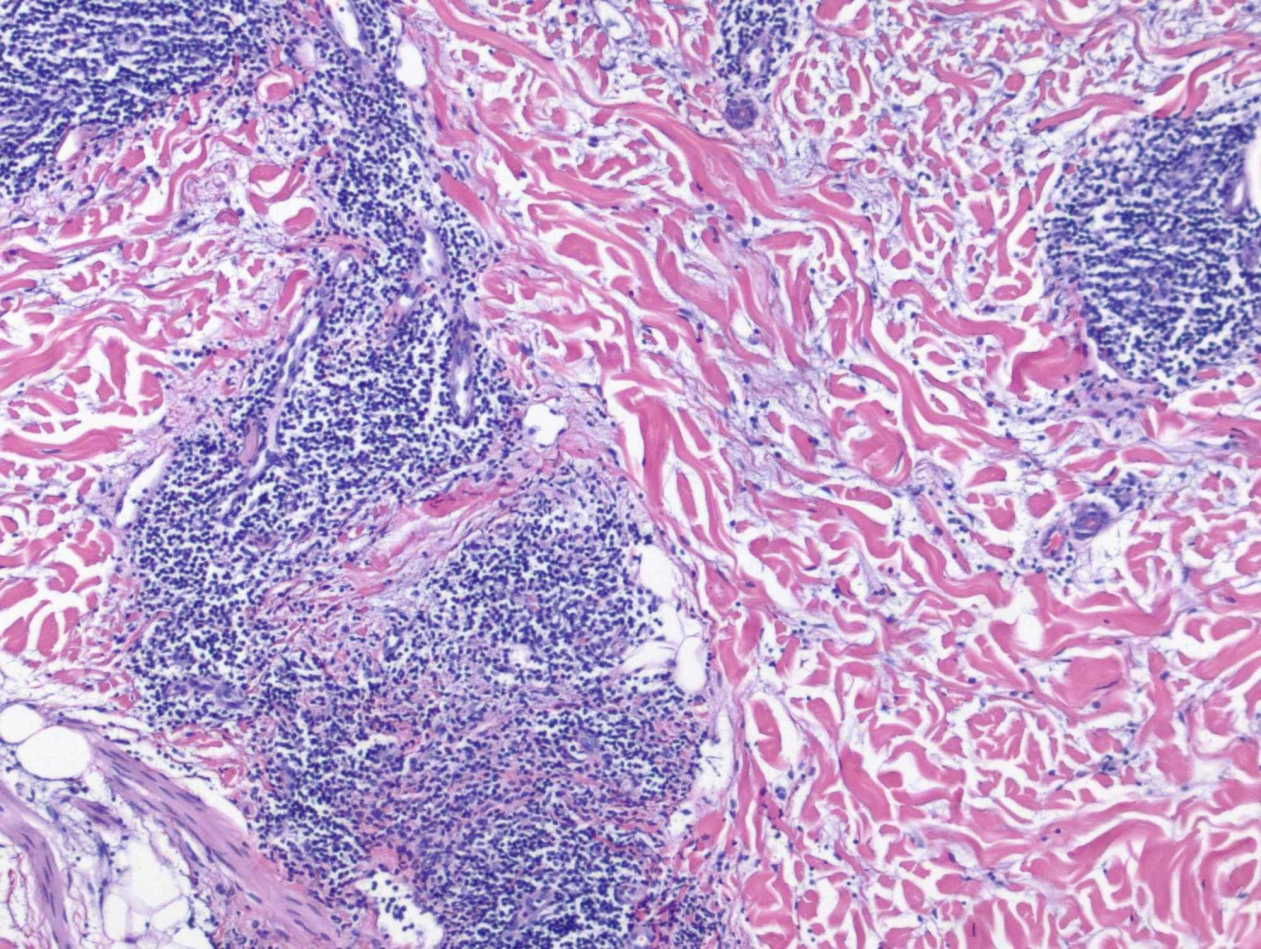
Hematoxylin and eosin stain of 4-mm punch biopsy (10x magnification) of lesion demonstrating deep perivascular and periadnexal lymphocytic infiltrate with dermal mucin deposition and edema.

**Figure 5:**
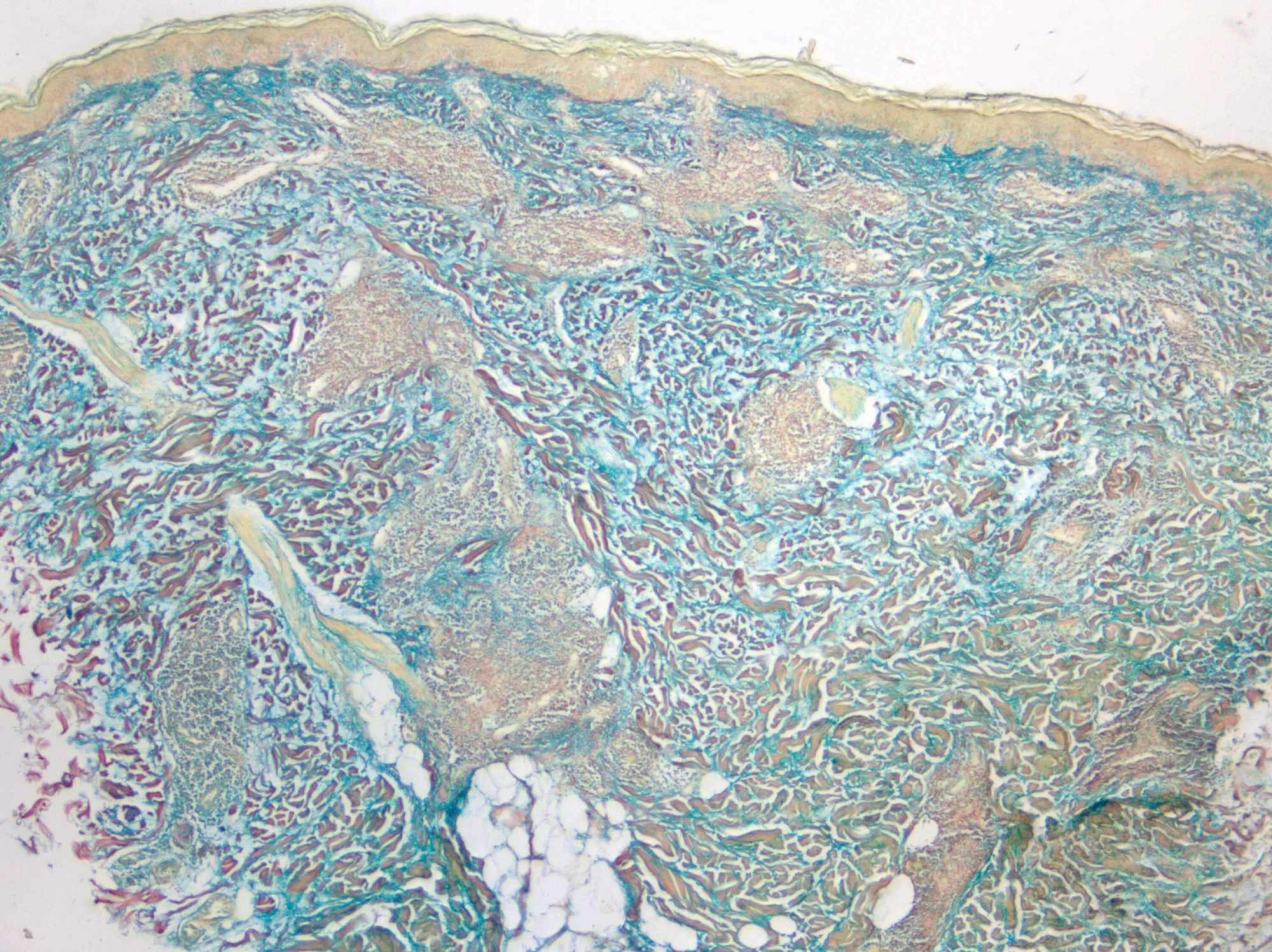
Colloidal iron stain of 4-mm punch biopsy (4x magnification) of lesion demonstrating excessive deposition of dermal mucin.

A 49-year-old Caucasian female with a past medical history of cutaneous squamous cell carcinoma and history of smoking tobacco use presented for evaluation of a pruritic rash on her back that had been present for approximately three months. She was clinically diagnosed with urticaria and initially treated with flurandrenolide 0.05% cream for two weeks with minimal improvement. Review of systems was noncontributory. Physical exam demonstrated several, well-demarcated, erythematous, and edematous plaques on the posterior trunk (Figures 1-2). Due to the refractory course of illness, other differential diagnoses were considered and the following diagnostics were ordered: complete metabolic panel, hepatitis panel, antineutrophil cytoplasmic antibodies, urinalysis, complement C1q antibody, C3 and C4, total complement, serum cryoglobulin IgA/IgG/IgM, erythrocyte sedimentation rate, nuclear antibody titer, and rheumatoid factor titer. Aside from an elevated total complement, >60 U/mL (reference range: 31-60 U/mL), all laboratory studies were within normal levels. Two 4-mm punch biopsies from lesions were taken from two different sites. They both demonstrated a superficial and deep perivascular and periadnexal lymphocytic infiltrate, dermal mucin, and edema (Figures 3-4). A colloidal iron immunohistochemical stain confirmed the excessive mucin deposition within the dermis (Figure 5). Given the clinical and histopathological correlation, a diagnosis of TLE was established. The patient was prescribed betamethasone dipropionate 0.05% spray and noted significant clinical improvement with near complete resolution at the follow-up visit, two weeks later.

## Discussion

Tumid lupus erythematosus was first documented by a German dermatologist Dr. Erich Hoffmann in 1909 [3]. TLE typically presents as annular, indurated, erythematous, and edematous plaques without epidermal involvement affecting the face and the trunk as those are typically sun-exposed sites [4]. In terms of epidemiology, one study identified an incidence of 16% among those patients diagnosed with cutaneous forms of lupus erythematosus [4]. The pathogenesis of TLE is currently unknown, but it is suspected that both genetic predispositions and environmental factors are involved in its development. Its association with autoimmune disease, specifically progression to systemic lupus erythematosus, is controversial due to the low prevalence of systemic lupus erythematosus, low index of serologic abnormalities, and relative absence of immunoglobulin deposition within affected skin lesions [1]. 

Unlike systemic lupus erythematosus and other variants of chronic cutaneous lupus erythematosus which predominantly affects females, tumid lupus does not have a high predilection for females. Males and females are at similar risk in developing this cutaneous process at any age, although male sex seemed to have an earlier average age of onset [5]. TLE is not limited to race and has been seen in individuals with darker skin types [6]. TLE is highly associated with ultraviolet light exposure as evidenced in Kuhn's study. It is also linked with smoking as well, which stands to reason as tobacco smoke is a known phototoxic agent [7].

Tumid lupus erythematosus is very loosely associated with systemic lupus erythematosus as serological testing such as antinuclear antibodies and double-stranded DNA antibody will be negative for the majority of TLE patients [4]. In Kuhn’s study only 10% of TLE patients had positive antinuclear antibodies, and one patient tested positive for the double-stranded DNA antibody [4]. Anti-Smith antibody titers, another marker specific for systemic lupus erythematosus should be considered in future serological testing of TLE to detect possible associations with systemic lupus erythematosus. However, this does not mean patients should forgo serological testing as there is still a small possibility of having a concurrent systemic lupus erythematosus [5, 8].

Tumid lupus erythematosus has been reported in patients with a history of TNF-α inhibitors and thiazide diuretics [9]. Some studies have shown TNF-α levels are increased in systemic lupus erythematosus patients, but had no correlation with disease activity [9]. Thiazide diuretics on the other hand have a more predictable effect on TLE development. Photosensitivity is a common side effect of thiazide diuretics and individuals treated with this therapy are at risk of developing TLE which is known to be a photosensitive condition [1]. These patients who develop this cutaneous condition may even require systemic corticosteroid therapy in addition to discontinuing thiazide diuretics to remit these lesions [10-11]. Other frequently used medications such as angiotensin converting enzyme (ACE) inhibitors and angiotensin II receptor blockers (ARBs) have been reported to induce TLE and lymphocytic infiltrate of Jessner-like lesions [11]. 

In the differential of TLE, the clinical and histopathological overlap of other similar dermatoses such as lymphocytic infiltrate of Jessner and reticular erythematous mucinosis needs to be considered. Similar to TLE, lymphocytic infiltrate of Jessner can present as nonscarring photosensitive plaques, but just previously discussed, lymphocytic infiltrate of Jessner does not have mucin within its composition [12]. Reticular erythematous mucinosis, however, does have a finding of dermal mucin deposition under histopathology. Reticular erythematous mucinosis and TLE have been considered to be on a spectrum of the same disease based on their similar clinical and histopathological findings [1]. Both conditions present with plaque-like lesions, are exacerbated by exposure to ultraviolet light, have an absence of several immune serological markers, and respond well to antimalarial therapy [12]. 

In our case, urticarial vasculitis was considered on the differential given the refractory nature of the urticarial-like plaques. However clinically, urticarial vasculitis typically presents with burning and pain rather than pruritus. Due to the absence of leukocytoclastic vasculitis on histology, the diagnosis of urticarial vasculitis was not favored. Furthermore, the laboratory studies ruled out hypocomplementemic vasculitis, which commonly presents with an elevated erythrocyte sedimentation rate, low serum C3 and C4, positive antinuclear antibodies, and anti-C1q antibodies [1]. 

Photoprotection, topical and/or intralesional corticosteroids, and topical calcineurin inhibitors are considered first line therapy [1]. For those who fail conservative therapy or have extensive disease, systemic treatment with antimalarials such as hydroxychloroquine or chloroquine should be utilized. Second line therapies include methotrexate or mycophenolate mofetil with folic acid supplementation. Third line therapies to consider if all other regimens fail include thalidomide or lenalidomide [4-5]. Pulse dye laser also remains a viable option for suppressive, noncurative therapy [13]. Trigger avoidance such as sun-exposure protection and avoidance of smoking are key components in preventing relapse of lesions in these patients.

## Conclusions

In conclusion, TLE is a rare clinical variant of chronic cutaneous lupus erythematosus presenting as photosensitive erythematous and edematous plaques with minimal progression to systemic lupus erythematosus. The lesions of TLE can be clinically indistinguishable from other dermal entities such as urticarial vasculitis and lymphocytic infiltrate of Jessner, thus requiring additional histological and laboratory studies. Clinicians should approach urticarial-like lesions with appropriate diagnostic and therapeutic modalities to provide optimal and timely patient outcomes. 
